# Surgical treatment of early-stage vulvar squamous cell carcinomas: a systematic review of guidelines for dermatologic surgeons

**DOI:** 10.1097/JW9.0000000000000104

**Published:** 2023-07-28

**Authors:** Elizabeth M. Rao, Caroline M. Wilkowski, Larissa DeSouza, Amy McNally, Jennifer Lucas

**Affiliations:** a Case Western Reserve University School of Medicine, Cleveland, Ohio; b Department of Dermatology, Dermatology and Plastic Surgery Institute, Cleveland Clinic, Cleveland, Ohio; c Department of Gynecologic Oncology, Minnesota Oncology, St. Paul, Minnesota

**Keywords:** gynecologic malignancies, SCC, vulvar

**What is known about this subject in regard to women and their families?**

- Vulvar squamous cell carcinomas are the most common of vulvar carcinomas. These tumors have the potential for significant morbidity and a great impact on the patients quality of life given their proximity to structures such as the clitoris, urethra, vagina, and anus. They may be treated by gynecologic surgeons, but they are also treated by dermatologic surgeons.

**What is new from this article as messages for women and their families?**

- We review guidelines from gynecology regarding the management of vulvar squamous cell carcinomas to inform dermatologic surgeons of reasons to escalate care. New directions for research are suggested given these guidelines and recent research on margin size.

Vulvar squamous cell carcinoma (vSCC) of the vulva represents approximately 90% of vulvar carcinomas. Proximity to structures such as the vagina, urethra, anus, and inguinofemoral lymph nodes increases vSCC morbidity significantly.^1^

Early-stage vSCC is considered appropriate for treatment by Mohs micrographic surgery (MMS), with advantageous intraoperative margin assessment.^2^ In gynecology, early-stage tumors are treated with wide local excision, radical vulvectomy, lymph node dissection, and adjuvant chemoradiation for specific cases. Understanding gynecologic-oncology recommendations aids in recognizing when to escalate treatment to provide optimal care. We provide a systematic review of the most up-to-date society guidelines for the treatment of early-stage vSCC to inform dermatologic surgeons caring for these patients.

A PubMed and Google Scholar search was performed using the terms “Vulvar” AND “Squamous cell carcinoma” AND “treatment” AND “guidelines” using Preferred Reporting Systems for Systematic Reviews and Meta-Analyses (PRISMA) guidelines. Of 341 initial search results, 18 articles remained after screening for updated versions and English language; of these, 13 related to surgical treatment of early-stage vulvar carcinomas and were included (Fig. 1).

**Fig. 1.**
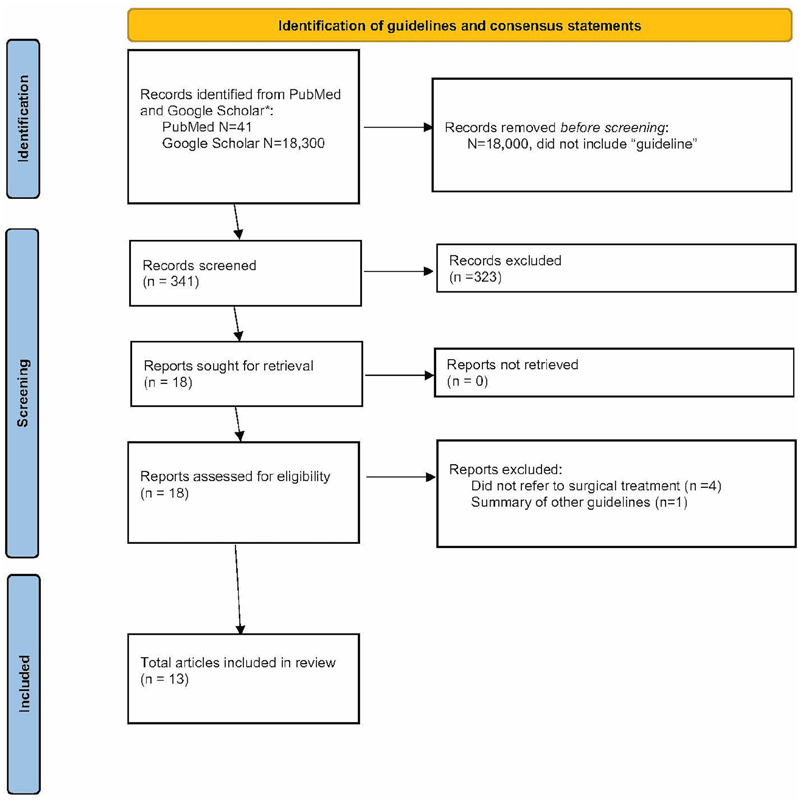
PRISMA diagram.

vSCC are staged by the Fédération internationale de gynécologie obstétrique (FIGO) 2021 classification and the American Joint Committee on Cancer staging.^3^ Early-stage tumors are FIGO stage IA-IB or American Joint Committee on Cancer stage TI-TII, and under 4 cm in size, without suspicion of nodal involvement.^3,4^ Tumors larger than 4 cm in diameter or involving local structures such as the urethra are considered locally advanced and may follow a different treatment paradigm.^4^ FIGO stage IA tumors are under 2 cm in diameter, with less than 1 mm stromal involvement; stage IB are those greater than 2 cm or with greater than 1 mm depth.

Many guidelines recommend FIGO stage IA, unifocal tumors undergo local excision with minimum 8 mm margins on fixed tissue; some societies recommend conservative 1–2 cm margins (Fig. 2). Guidelines from the German Society for Gynecology and Obstetrics and German Cancer Society state that margins of 3 mm measured histologically may be acceptable.^5^ Adjuvant radiation therapy should be considered for diffuse disease, deep invasion (>1 mm), or close margins (<8 mm). For residual tumors, guidelines recommend reexcision or radiation therapy for unresectable tumors. For tumors invading deeper than 1 mm and extending greater than 2 cm, guidelines recommend escalation to radical vulvectomy or radical local excision.

**Fig. 2.**
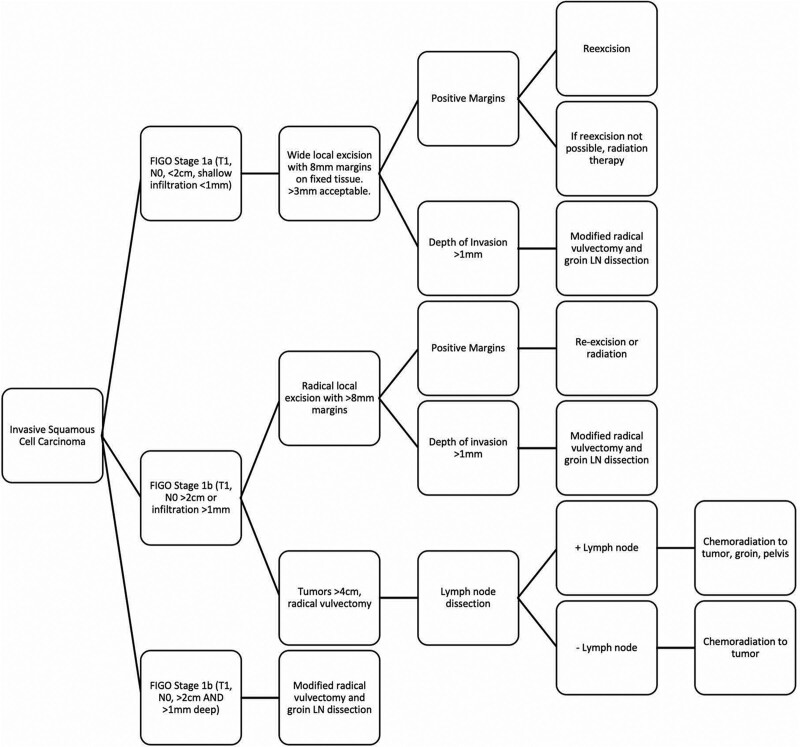
Summary of recommendations for treatment of vSCC after discovering a suspect lesion. If clear margins on fixed tissue, follow up every 6 months for 5 years, and every year thereafter. FIGO, Fédération internationale de gynécologie obstétrique; vSCC, vulvar squamous cell carcinoma.

Lymph node dissection via sentinel lymph node biopsy or inguinofemoral node dissection is recommended for FIGO stage IB and higher or depth of invasion >1 mm. Lateral tumors (>1 cm from midline) should undergo ipsilateral unilateral lymph node biopsy. Midline lesions should undergo bilateral lymph node biopsy.

Dermatologists should be aware of the guidelines utilized by other specialties treating vSCC due to the morbidity associated with the disease in this region. Guideline-based vSCC treatment includes faster escalation to radical vulvectomy and lymph node dissection than other high-risk sites treated by MMS such as facial mask areas. More narrow margins of >3 mm on fixed tissue may be acceptable.^5^ Further research on margin size in vSCC may inform treatment focused on tissue conservation or intraoperative margin assessment, hallmarks of MMS. Limited data currently exists on recurrence rates for vSCC treated by MMS; future research may inform evolving guidelines in treatment.

## Conflicts of interest

None.

## Funding

None.

## Study approval

This study did not require patient or protected health information and therefore did not require IRB approval.

## Author contributions

EMR and CW: Participated in research design, writing of the article, and the performance of the research. LD: Participated in the writing of the article and the performance of the research. AM and JL: Participated in research design and writing of the article.
